# Asylum Workers

**Published:** 1916-12-02

**Authors:** 


### THE EDITOR'S LETTER-BOX.

### Asylum Workers.

*To the Editor of* The Hospital.

Sir,—Very few philanthropic subjects, except those in direct touch with the war, are interesting to the public to-day. There is, however, both directly and indirectly, a war work which receives scant attention. We allude to that on behalf of the wounded in mind; not only to victims of war shock (who are not necessarily insane at all), but to those who have for years filled our mental hospitals. We have heard much of special hospitals for the wounded in mind; but very little of those who have for years worked as mental specialists, chaplains, nurses, and attendants, and are now submitting to overwork and overcrowding through war conditions. We owe, therefore, a double debt of gratitude to any legitimately formed Association on behalf of those who are too busy to attend to their own wide interests. Years ago the Asylum Workers' Association was founded by Miss Honnor Morten, a lady well known in the world of general nursing, and Miss Laura Evans, matron of the Northampton County Asylum, with the assistance of Dr. Harding, medical superintendent of that institution. Subsequently Dr. G. E. Shuttleworth, the retired superintendent of the Royal Albert Institution for Mental Defectives at Lancaster, accepted the post of hon. secretary, which he has held for twenty years; and it is supported by all the most prominent mental specialists of our time. It works away in war-time as in peace. It is one of the watch-dogs of our country. It has not only a great band of vice-presidents, but it calls on the humblest member to do his or her best in practically promoting the welfare of those afflicted in mind.

What, then, does the Association stand for? Briefly, the raising of the status of the asylum worker by all means in its power. In prehistoric days the mental nurse was despised as of inferior caste. The improvement in his or her position is due mainly to the exertions of the Asylum Workers' Association, many of whose members are also members of the Medico-Psychological Association, which has set a uniform and satisfactory standard for the training and examination of mental nurses all over the Empire.

A great deal remains yet to be done to place the worker in his or her true position in the eyes of the world, and to ensure that he or she is adequately recognised and represented in the councils of those who deal with the nursing profession as a whole. The Association is therefore ever watching jealously the interests of the asylum worker both in Parliament and elsewhere. It has been successful in securing assured and adequate pensions for mental nurses in public asylums. It has an organ—the *Asylum News*—in which not only may the views of practical workers be aired, but many useful and instructive articles have appeared. The Convalescent Fund, founded by the Association for asylum workers suffering in health, often from the strain of their duties, is flourishing. The Association is as careful of the great medical and nursing traditions of the past as determined to maintain them in the future. It is not run on "trades union" lines, nor will it concoct strikes if it does not get exactly what it likes all at once. It proclaims that asylum is not a trade but a vocation founded on the basis of Christian charity, a profession and an art calling for the highest qualities of heart and head. In order to develop new activities a larger register of members is desirable. Unity is strength, the rank and file are wanted to "do their bit," their aims and aspirations should be expressed through their local secretaries. It may not be possible to initiate new efforts in war-time, but it is possible to recruit new members, to repair defences, and to carry on till peace liberates us for a more vigorous battle which will be fought in many other places than "somewhere in France." The administration of the Mental Deficiency Act of 1913, though curtailed through the exigencies of war, will probably bring into the scope of institution work some hundred thousand additional persons requiring kindly care and control. This fact alone points out clearly the heavy responsibilities of the asylum worker. Mr. J. B. Wilson, assistant secretary, 8 Lancaster Place, Hampstead, will gladly forward particulars.—Yours faithfully,

B. J.

London, November 21, 1916.

